# Regression of Bilateral Choroidal Metastases From Primary Lung Carcinoma in Response to Systemic Bevacizumab and Platinum Doublet

**DOI:** 10.7759/cureus.76900

**Published:** 2025-01-04

**Authors:** Vaibhav Bhatt, Shweta Parakh, Shrutanjoy Das, Abhijaat Chaturvedi, Suhani Malhotra, Vishali Gupta, Rupesh Agrawal, Gaurav Luthra, Saurabh Luthra

**Affiliations:** 1 Ophthalmology, Drishti Eye Institute, Dehradun, IND; 2 Ophthalmology, Postgraduate Institute of Medical Education and Research, Chandigarh, IND; 3 Ophthalmology, Tan Tock Seng Hospital, Singapore, SGP

**Keywords:** bevacizumab, carcinoma lung, choroidal metastases, multimodal ophthalmic imaging, platinum based chemotherapy

## Abstract

A 58-year-old male presented with large yellowish choroidal mass lesions with exudative subretinal fluid (SRF) in both eyes. A computed tomography (CT) scan of the thorax revealed a mass lesion in the apical segment of the right upper lobe. Fine needle aspiration cytology (FNAC) of right supraclavicular lymph nodes revealed metastatic mucinous adenocarcinoma. Positron emission tomography-computed tomography scan (PET-CT) showed multiple lymphatic, pulmonary, and skeletal metastases. After six cycles of chemotherapy (platinum doublet and systemic bevacizumab), clinical examination and multimodal imaging showed complete regression of the choroidal lesions. Ours is the first case report of bilateral choroidal metastases from primary pulmonary adenocarcinoma (possibly arising from hazardous occupational exposure in the sericulture industry) that showed complete regression in response to standalone systemic chemotherapy agents, platinum doublet and systemic bevacizumab.

## Introduction

The uveal tract is the most common site for metastases affecting the choroid (88%), ciliary body (2%), and iris (9%) [1,2]. Breast and lung carcinoma are the predominant tumors to metastasize to the uvea [2]. The reported incidence of ocular metastases from lung carcinoma is 2 to 7% [3]. Ocular symptoms like visual impairment can be an initial presentation and alert the clinician to the presence of primary carcinoma [4]. Treatment modalities for choroidal metastases include external beam radiotherapy, plaque radiotherapy, transpupillary thermotherapy, surgical resection, and intravitreal chemotherapy [5]. Administration of systemic chemotherapy with or without targeted local therapy for choroidal metastases from lung cancer has also shown favourable results [5,6].

We report a unique case of bilateral choroidal metastases as the primary feature of primary mucinous pulmonary adenocarcinoma in a male non-smoker, possibly arising from hazardous occupational exposure in the sericulture industry. The patient showed complete regression of choroidal metastases in response to systemic bevacizumab and chemotherapy (platinum doublet).

## Case presentation

**Figure 1:**
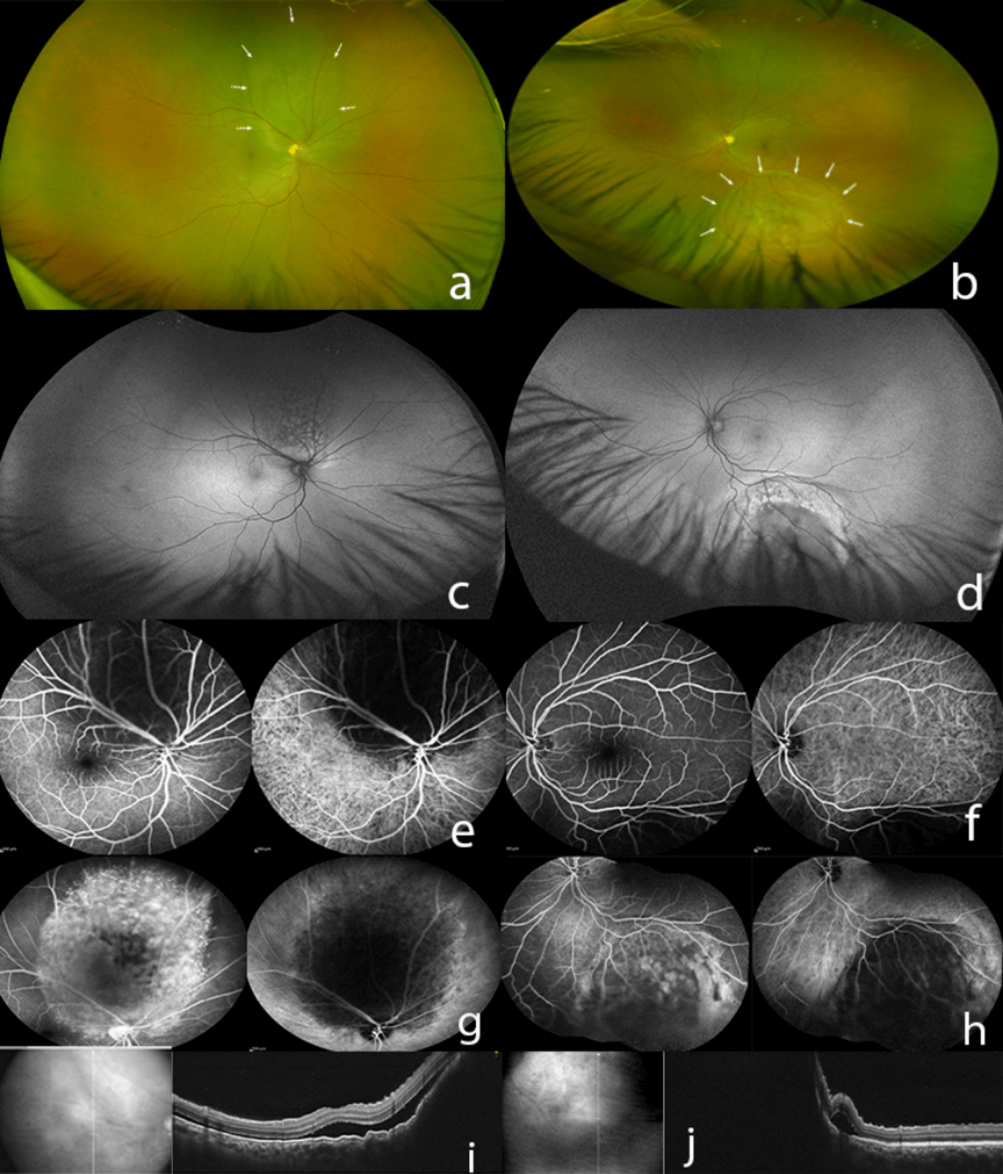
Multimodal imaging of choroidal metastases in both eyes from primary lung carcinoma. At the first visit: (a, b) Ultra-widefield fundus image of right and left eye respectively showing large yellowish choroidal mass lesion with overlying subretinal fluid (SRF) (white arrows) in superior midperiphery encroaching the macula and the inferior midperiphery. (c, d) Ultra-widefield fundus autofluorescence image both eyes (OU) showing central hypoautofluorescence within the choroidal mass lesion surrounded by hyperautofluorescence. (e, f) FFA and ICGA showing hypofluorescence and hypocyanescence within the mass lesion OU. (g, h) Late-phase FFA shows intense leakage at the edges of mass lesion OU, and late-phase ICGA continues to show hypocyanescence OU. (i, j) SD-OCT through the lesions demonstrating a large elevated choroidal hyporeflective lesion, with “lumpy bumpy” overlying RPE and overlying subretinal fluid with elongated shaggy photoreceptors OU. OS: oculus sinister; OD: oculus dexter; OU: oculus uterque; FFA: fundus fluorescein angiography; ICGA: indocyanine green angiography; SD-OCT: spectral-domain optical coherence tomography; RPE: retinal pigment epithelium.

A 58-year-old male, non-smoker, working in the sericulture industry, presented with painless, gradually progressive diminution of vision in the right eye, oculus dexter (OD), for one month. The best corrected visual acuity (BCVA) was 6/18 OD and 6/6 in the left eye, oculus sinister (OS). Intraocular pressure was normal (12 mm Hg) in both eyes, oculus uterque (OU). Anterior segment examination was unremarkable OU. Fundus examination and ultra-widefield imaging (Optos PLC, Dunfermline, United Kingdom) revealed OD, a large yellowish choroidal mass lesion in the superior midperiphery encroaching the macula, with overlying subretinal fluid (SRF) (Figure 1a, Figure 1c). A similar macula-sparing large yellowish choroidal mass lesion with subretinal fluid (SRF) was noted in the inferior mid-periphery OS (Figure 1b, Figure 1d). Fundus fluorescein angiography (FFA) (Heidelberg Retina Angiograph, Heidelberg Engineering, Heidelberg, Germany) showed early hypofluorescence within the mass lesion and intense leakage at the edges of mass lesion in the late phase OU (Figure 1e, Figure 1f, Figure 1g, Figure 1h). Indocyanine green angiography (ICGA) (Heidelberg Retina Angiograph, Heidelberg Engineering, Heidelberg, Germany) showed early and late hypocyanescence within the mass lesion OU (Figure 1e, Figure 1h). Spectral-domain optical coherence tomography (SD-OCT) (RTVue XR Avanti, Optovue Inc., Fremont, CA, USA) through the lesions revealed a large choroidal elevation with retinal pigment epithelium (RPE) undulation and SRF (Figure 1i, Figure 1j).

**Figure 2:**
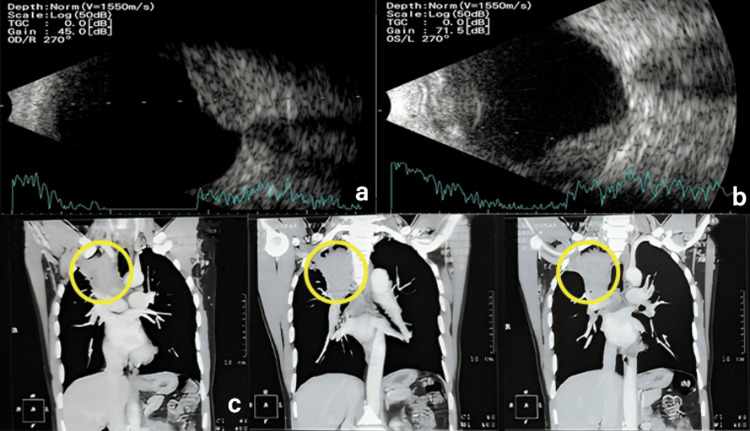
At presentation. (a, b) B-scan ultrasound showing a diffuse homogenous mass lesion involving the retinochoroidal complex with subretinal fluid and medium internal reflectivity OU. (c) Coronal section of CT chest showing heterogeneously enhancing soft tissue density mass lesion (yellow circles) seen in the apical segment of right upper lobe extending up to the right hilar region. OS: oculus sinister; OD: oculus dexter; OU: oculus uterque.

Notably, choroidal vasculature details were obscured, and photoreceptors showed a typical “shaggy” appearance. B-scan ultrasound (Nidek Co. Ltd, Aichi, Japan) showed a diffuse homogenous mass lesion involving the retinochoroidal complex with subretinal fluid and medium internal reflectivity OU (Figure 2a, Figure 2b). T-sign was absent, and no choroidal excavation or retinochoroidal calcification was noted. Based on the above clinical signs and multimodal imaging features, a provisional diagnosis of bilateral choroidal metastases was made. A thorough systemic evaluation (including complete blood count, general blood picture, computed tomography scan of thorax, and ultrasound of abdomen and pelvis) to search for a possible primary carcinoma was performed. A computed tomography (CT) scan of the thorax revealed a mass lesion in the apical segment of the right upper lobe with a heterogeneous spiculated margin, encasing the right superior pulmonary artery and causing luminal narrowing (Figure 2c). Given a probable lung carcinoma, the patient was urgently referred to an oncologist.

The patient underwent ultrasonography (USG) guided fine needle aspiration cytology (FNAC) of right supraclavicular lymph nodes, which revealed metastatic mucinous adenocarcinoma. Positron emission tomography and computed tomography (PET-CT) showed multiple fluorodeoxyglucose (FDG) avid lymphatic, pulmonary and skeletal metastases. A diagnosis of Stage 4b non-small cell lung carcinoma (mucinous adenocarcinoma) was confirmed. Systemic palliative chemotherapy was initiated in the form of intravenous paclitaxel (300 mg), intravenous carboplatin (520 mg), and intravenous bevacizumab (500 mg) every three to four weeks for six cycles (induction).

**Figure 3:**
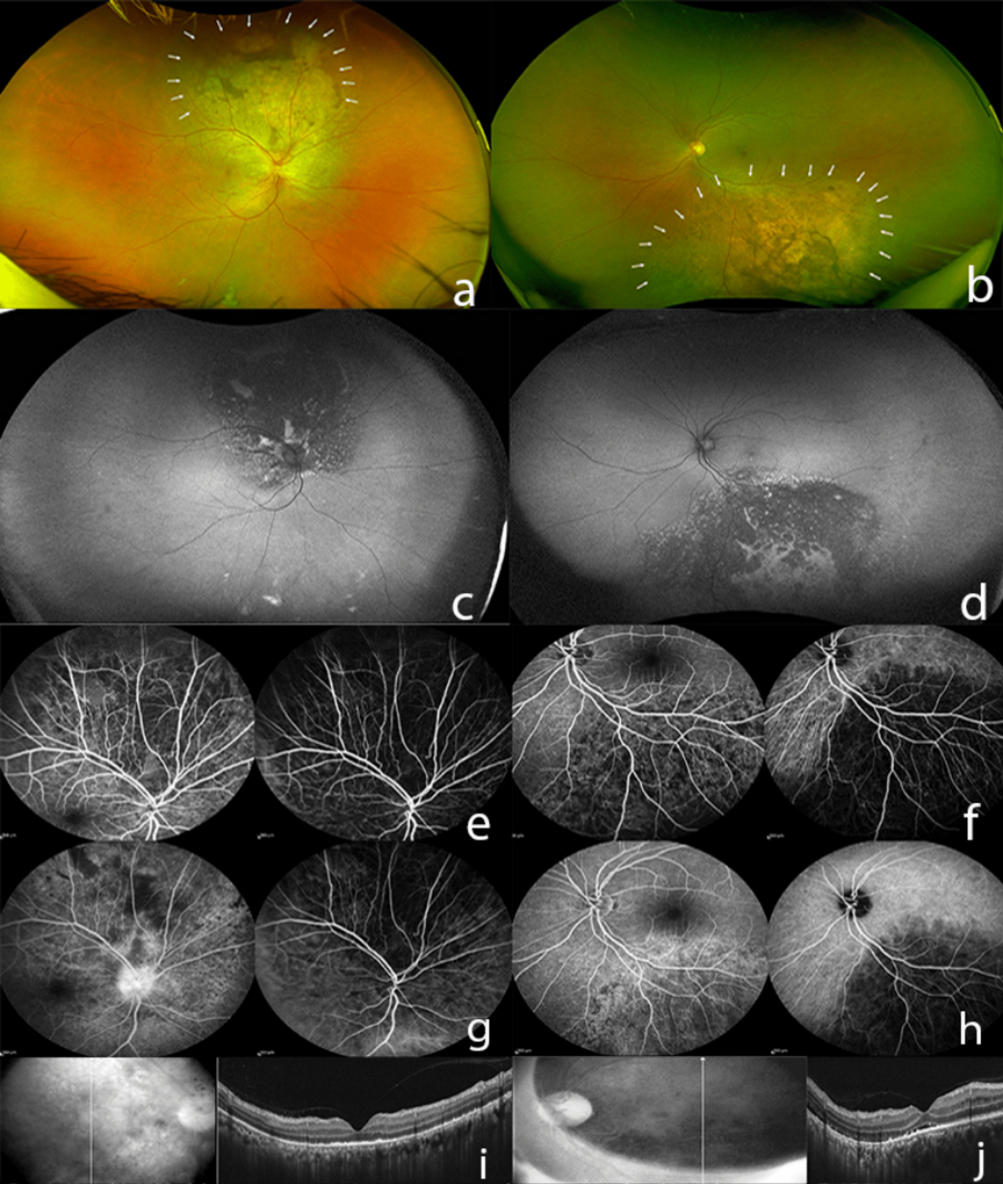
At the 20-week follow-up visit (post 6 cycles of palliative chemotherapy with platinum doublet and systemic bevacizumab): (a, b) Ultra-widefield fundus image of both eyes (OU) showing flattening of choroidal lesion and alteration in surface pigmentation (white arrows). (c, d) Ultra-widefield fundus autofluorescence image OU showing prominent stippled hypoautofluorescence and hyperautofluorescence. (e, f) FFA and ICGA show normalization of hypofluorescence and hypocyanescence within the mass lesion noted previously OU. (g, h) Late-phase FFA shows stippled hyperfluorescence OU and late-phase ICGA shows normalization of previously noted hypocyanescence OU. (i, j) SD-OCT through the lesions illustrating regression of the choroidal hyporeflective lesion with resolution of overlying subretinal fluid OU. OS: oculus sinister; OD: oculus dexter; OU: oculus uterque; FFA: fundus fluorescein angiography; ICGA: indocyanine green angiography; SD-OCT: spectral-domain optical coherence tomography.

After six cycles of chemotherapy (at the 20-week follow-up), fundus examination and ultra-widefield imaging showed complete regression of choroidal lesion OU (Figure 3a, Figure 3b, Figure 3c, Figure 3d). BCVA improved to 6/9 OD and was maintained at 6/6 OS. Early phase fundus fluorescein angiography (FFA) and indocyanine green angiography (ICGA) showed normalization of hypofluorescence and hypocyanescence within the mass lesion noted OU (Figure 3e, Figure 3f) previously. Late-phase FFA showed stippled hyperfluorescence OU, and late-phase ICGA revealed normalization of previously noted hypocyanescence OU (Figure 3g, Figure 3h). SD-OCT demonstrated complete regression of choroidal elevation and SRF OU (Figure 3i, Figure 3j).

**Figure 4:**
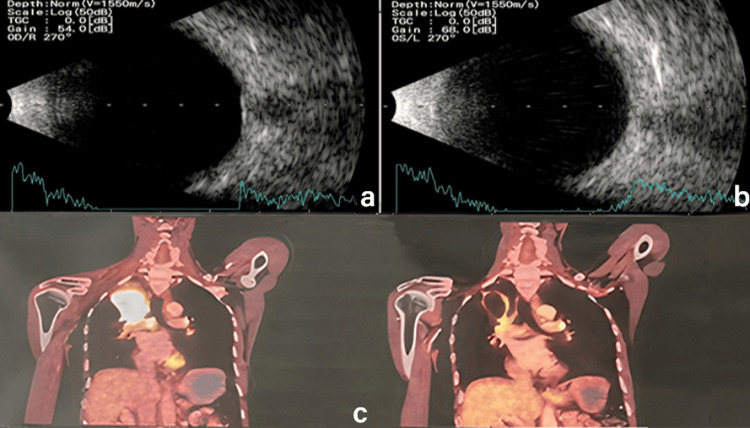
At 20-week follow-up. (a, b) B-scan ultrasound showing regression of mass lesion with a resolution of overlying subretinal fluid OU. (c) PET-CT scan showing residual lymphatic and primary pulmonary malignant pathology and regressed skeletal metastases. OU: oculus uterque; PET-CT: Positron Emission Tomography and Computed Tomography.

B-scan USG showed complete regression of the subretinal mass lesion OU (Figure 4a, Figure 4b). PET-CT scan revealed residual lymphatic and primary pulmonary malignant pathology and regressed skeletal metastases (Figure 4c). The patient was further advised for maintenance chemotherapy in the form of systemic bevacizumab. Unfortunately, he succumbed to systemic complications after the last cycle of chemotherapy at the end of seven months.

Table 1 shows a comparison of multimodal imaging features at presentation and at 20-week follow-up. 

**Table 1:** Comparison of multimodal imaging features at presentation and at 20-week follow-up. OS: oculus sinister; OD: oculus dexter; OU: oculus uterque; FFA: fundus fluorescein angiography; ICGA: indocyanine green angiography; SD-OCT: spectral-domain optical coherence tomography.

Images	At presentation	At 20-week follow-up
Ultrawide field image (Colour image)	Ultra-widefield fundus images of the right and left eye, respectively, show a large yellowish choroidal mass lesion with overlying subretinal fluid (SRF) (white arrows) in the superior mid periphery encroaching the macula and the inferior mid periphery (1a, 1b).	Ultra-widefield fundus image of both eyes (OU) showing flattening of choroidal lesion and alteration in surface pigmentation (white arrows) (3a, 3b).
Ultrawide field image (Autofluorescence)	Central hypoautofluorescence within the choroidal mass lesion surrounded by hyperautofluorescence (1c, 1d).	Prominent stippled hypoautofluorescence and hyperautofluorescence (3c, 3d).
Early phase FFA and ICGA	Hypofluorescence and hypocyanescence within the mass lesion OU (1e, 1f).	Hypofluorescence and hypocyanescence within the mass lesion were noted OU (3e, 3f) previously.
Late phase FFA and ICGA	Late-phase FFA showing intense leakage at the edges of mass lesion OU and late-phase ICGA continues to show hypocyanescence OU (1g, 1h)	Late-phase FFA shows stippled hyperfluorescence OU and late-phase ICGA shows normalization of previously noted hypocyanescence OU. (3g, 3h)
SD-OCT	Spectral-domain optical coherence tomography (SD-OCT) through the lesions demonstrated a large elevated choroidal hyporeflective lesion, with “lumpy bumpy” overlying retinal pigment epithelium (RPE) and overlying subretinal fluid with elongated shaggy photoreceptors OU (1i, 1j)	Spectral-domain optical coherence tomography (SD-OCT) through the lesions shows regression of the choroidal hyporeflective lesion with resolution of overlying subretinal fluid OU (3i, 3j).
B-scan ultrasound	Diffuse homogenous mass lesion involving the retinochoroidal complex with subretinal fluid and medium internal reflectivity OU (2a, 2b).	Regression of mass lesion with resolution of overlying subretinal fluid OU (4a, 4b).

## Discussion

Due to its rich arterial supply, the choroid (88%) is one of the more common sites for metastases in the eye, followed by the iris (9%) and ciliary body (2%). In the case of breast carcinoma, chances of metastases to choroid and orbit are almost equal, but lung carcinoma has more preponderance to choroid than orbit [1,5]. Systemic cancer is usually diagnosed before choroidal metastasis can be detected [7]. Patients with lung cancer have lower survival rates, which contributes to lower rates of choroidal metastasis from primary lung carcinoma [8]. Bilateral choroidal metastases as a presenting feature of lung carcinoma are a rare manifestation and have been reported in only a few cases [9].

Differential diagnoses include choroidal hemangioma, amelanotic choroidal melanoma, posterior scleritis, choroidal osteoma, and other lesions that can be ruled out with the aid of multimodal imaging [5]. OCT has certain pathognomonic features, such as speckled/shaggy photoreceptors, subretinal fluid, and RPE undulation with thickening and obscured choroidal vessel architecture - all noted in our patient [10]. Characteristic B-scan findings are moderate echogenic subretinal mass with indistinct borders with overlying exudative retinal detachment and moderate sound attenuation [11].

The decision to pursue local therapy for choroidal metastases is multidisciplinary and involves the patient, oncologist, and ophthalmologist. Important factors include patient preference, overall health, location and extent of intraocular lesion, and visual symptoms. Choroidal metastatic lesions responding well to systemic therapeutic modalities generally do not require direct local treatment; in cases where a choroidal metastasis is found to be enlarging during/in spite of systemic therapy or is the only distant site for metastasis, local therapy to the ocular tissues is recommended [12,13,14]. Local therapy modalities can include radiotherapy (external beam, plaque brachytherapy, gamma knife, and proton beam), intravitreal injection, photodynamic therapy, laser therapy, cryotherapy, or resection. The goal of emerging local therapies is to restore or maintain visual function while minimizing ocular toxicity, described as collateral damage to other parts of the eye. Palliative treatment for choroidal metastases includes external beam radiotherapy, transpupillary thermotherapy, or enucleation [5]. External beam radiotherapy in the range of 20-50 Gy can be used to control tumor growth for large tumors involving optic nerve/macula and provide symptomatic relief. Visual restoration after external beam radiotherapy is usually short-lived due to complications like cataract formation and radiation retinopathy [15]. Few case reports have documented choroidal metastases from non-small cell lung carcinoma that can be efficiently managed with systemic chemotherapy [16,17]. Bevacizumab, an anti-VEGF agent, has been used systemically [6] or intravitreally [18-20] in managing choroidal metastases as an alternative chemotherapy option. These reports showed the anti-permeability and anti-angiogenic effects of bevacizumab on new tumor vessels. Systemic administration of bevacizumab reportedly produces more active drug concentrations at the borders of the lesion owing to the abundant choroidal blood supply [5]. As per American College of Chest Physicians Evidence-Based Clinical Practice Guidelines, bevacizumab improves survival when combined with carboplatin and paclitaxel in a clinically selected subset of patients with stage IV NSCLC and good performance status (PS) (nonsquamous histology, lack of brain metastases, and no hemoptysis). In these patients, the addition of bevacizumab to carboplatin and paclitaxel is recommended [21]. Based on these recommendations, our patient received systemic palliative chemotherapy in the form of paclitaxel, carboplatin, and intravenous bevacizumab.

Our patient was a non-smoker working in the sericulture industry. Trivalent chromium, used to fix silk dyes, undergoes oxidation into hexavalent chromium. Hexavalent chromium compounds [Cr(VI) compounds] are classified as human carcinogens by the International Agency for Research on Cancer (IARC) of the World Health Organization (WHO) based on epidemiological studies linking Cr(VI) to pulmonary carcinoma [22]. Halasova et al. have documented occupational exposure to chromium as an important risk factor for lung cancer and reported a higher number of small-cell lung carcinomas in exposed individuals [23]. During Cr(VI) reduction, a wide range of genetic lesions are generated, including Cr-DNA binary (mono) adducts, Cr-DNA ternary adducts, DNA protein cross-links, bi-functional (DNA inter-strand cross-links) adducts, single-strand breaks, and oxidized bases. Cr(VI) exposure causes a classical DNA damage response within cells, including activation of the p53 signaling pathway and cell cycle arrest or apoptosis [24]. The role of potential carcinogens in the sericulture industry warrants further research so that appropriate safety measures can be taken at the workplace. A few cases reported by George et al. [6] and Kourie et al. [5] have highlighted the efficacy of platinum doublet with systemic bevacizumab in achieving complete regression of unilateral choroidal metastases from poorly differentiated large-cell lung carcinoma and pulmonary adenocarcinoma respectively. To the best of our knowledge, this is the first reported case of bilateral choroidal metastases from primary pulmonary adenocarcinoma that showed complete regression in response to standalone systemic chemotherapy agents- platinum doublet and systemic bevacizumab.

## Conclusions

Seemingly innocuous ocular signs and symptoms such as mild ptosis, mild proptosis, strabismus, conjunctival hyperemia, neurosensory retinal detachment, decreased vision, visual field defects, floaters, and pain can be the presenting clinical features of an underlying ominous systemic malignancy. The clinician should be aware of potential occupational hazards in patients with no other obvious risk factors. Systemic chemotherapy alone can lead to complete resolution of choroidal metastases. Long-term follow-up is required in these cases to assess treatment response and possible need for local therapy. In addition, the role of potential carcinogens in the sericulture industry warrants further research to establish a causal association. This may help in the institution of appropriate prophylactic measures for those at risk.
